# Printed educational messages fail to increase use of thiazides as first-line medication for hypertension in primary care: a cluster randomized controlled trial [ISRCTN72772651]

**DOI:** 10.1186/s13012-016-0486-3

**Published:** 2016-09-17

**Authors:** Merrick Zwarenstein, Jeremy M. Grimshaw, Justin Presseau, Jill J. Francis, Gaston Godin, Marie Johnston, Martin P. Eccles, Jacqueline Tetroe, Susan K. Shiller, Ruth Croxford, Diane Kelsall, J. Michael Paterson, Peter C. Austin, Karen Tu, Lingsong Yun, Janet E. Hux

**Affiliations:** 1 Centre for Studies in Family Medicine, Department of Family Medicine, Schulich School of Medicine and Dentistry, Western University, 1465 Richmond Street, London, Ontario N6A 3K7 Canada; 2 Institute for Clinical Evaluative Sciences, 2075 Bayview Avenue, Toronto, Ontario M4N 3M5 Canada; 3 Ottawa Hospital Research Institute, The Ottawa Hospital—General Campus, 501 Smyth Road, Box 711, Ottawa, Ontario K1H 8L6 Canada; 4 Faculty of Medicine, University of Ottawa, 451 Smyth Road, Ottawa, Ontario K1H 8M5 Canada; 5 School of Epidemiology, Public Health and Preventive Medicine, 451 Smyth Road, Ottawa, Ontario K1H 8M5 Canada; 6 School of Health Sciences, City University London, Northampton Square, London, EC1V 0HB UK; 7 Faculty of Nursing, Laval University, Pavillon Ferdinand-Vandry, 1050 Avenue de la Medicine, Room 1445, Quebec City, Quebec G1V 0A6 Canada; 8 Institute of Applied Health Sciences, College of Life Sciences and Medicine, University of Aberdeen, 2nd floor, Health Sciences Building, Foresterhill, Aberdeen, AB25 2ZD UK; 9 Institute of Health and Society, Newcastle University, Baddiley-Clark Building, Richardson Road, Newcastle Upon Tyne, NE2 4AX UK; 10 Retired, Ottawa, Canada; 11 Canadian Medical Association Journal, 1867 Alta Vista Drive, Ottawa, Ontario K1G 5W8 Canada; 12 Institute of Health Policy, Management and Evaluation, University of Toronto, Health Sciences Building, 155 College Street, Suite 425, Toronto, Ontario M5T 3M6 Canada; 13 Faculty of Medicine, University of Toronto, 1 King’s College Circle, Medical Sciences Building, Toronto, Ontario M5S 1A8 Canada; 14 Canadian Diabetes Association, 522 University Ave, Toronto, Ontario M5G 2A2 Canada

## Abstract

**Background:**

Evidence on the effectiveness of printed educational messages in contributing to increasing evidence-based clinical practice is contradictory. Nonetheless, these messages flood physician offices, in an attempt to promote treatments that can reduce costs while improving patient outcomes.

This study evaluated the ability of printed educational messages to promote the choice of thiazides as the first-line treatment for individuals newly diagnosed with hypertension, a practice supported by good evidence and included in guidelines, and one which could reduce costs to the health care system.

**Methods:**

The study uses a pragmatic, cluster randomized controlled trial (randomized by physician practice group).

**Setting:**

The setting involves all Ontario general/family practice physicians.

Messages advising the use of thiazides as the first-line treatment of hypertension were mailed to each physician in conjunction with a widely read professional newsletter. Physicians were randomized to receive differing versions of printed educational messages: an “insert” (two-page evidence-based article) and/or one of two different versions of an “outsert” (short, directive message stapled to the outside of the newsletter). One outsert was developed without an explicit theory and one with messages developed targeting factors from the theory of planned behaviour or neither (newsletter only, with no mention of thiazides).

The percentage of patients aged over 65 and newly diagnosed with hypertension who were prescribed a thiazide as the sole initial prescription medication. The effect of the intervention was estimated using a logistic regression model estimated using generalized estimating equation methods to account for the clustering of patients within physician practices.

**Results:**

Four thousand five hundred four physicians (with 23,508 patients) were randomized, providing 97 % power to detect a 5 % absolute increase in prescription of thiazides. No intervention effect was detected. Thiazides were prescribed to 27.6 % of the patients who saw control physicians, 27.4 % for the insert, 26.8 % for the outsert and 28.3 % of the patients who saw insert + outsert physicians, *p* = 0.54.

**Conclusions:**

The study conclusively failed to demonstrate any impact of the printed educational messages on increasing prescribing of thiazide diuretics for first-line management of hypertension.

**Trial registration:**

ISRCTN72772651

**Electronic supplementary material:**

The online version of this article (doi:10.1186/s13012-016-0486-3) contains supplementary material, which is available to authorized users.

## Background

The cost-effectiveness of treatment options, particularly for the treatment of common health conditions, can have a large impact on the cost of health care. At the time of the conduct of the present trial, over a third of Canadians between the ages of 18 and 74 years had hypertension [1]; and despite the fact that only a fraction of these individuals were being treated [2], prescription drugs used in the treatment of hypertension are the leading therapeutic category of prescription drugs in Canada, accounting for 20 % of total prescription drug sales [3]. Decisions concerning the medical management of hypertension therefore have the potential to have a significant impact on health care costs.

Initial treatment of hypertension with thiazide diuretics has been shown to significantly reduce morbidity and mortality, with benefits at least as great as with other classes of antihypertensive drugs, at less cost [4]. Yet in Canada and elsewhere, there is evidence of low adherence to published recommendations that thiazides be used as the first-line treatment for patients with uncomplicated hypertension [2]. The cost to patients and insurers may be high: one study estimated savings of $13.8 million (in US dollars in the year 2000) for Canada (year 2000 population 31,281,100) by using thiazides as the first-line antihypertensive drug [2]. Since most individuals with hypertension were not being treated [2, 5], more aggressive screening and treatment of hypertension would markedly increase the number of prescriptions and thus the beneficial economic impact of choosing thiazides over other antihypertensive treatment.

Printed educational messages (PEMs) directed to physicians are one way to address health care gaps that are under physician influence. While PEMs have the advantages of low cost and easy dissemination, there is great uncertainty about their effects. Several early systematic reviews concluded that printed materials, on their own, do not lead to change in physician practices [6, 7]. However, Grimshaw observed that the median effect in the RCTs where guidelines were disseminated as PEMs was 8.1 % absolute risk reduction (range +3.6 to +17.0 %) [8], on par with other much more expensive interventions like audit and feedback or academic outreach, and this result was the main stimulus for the current trial. A subsequent larger review of PEMs observed a smaller effect size, showing a median absolute risk difference of 0.02 (range 0 to 0.11) in seven RCTs reporting categorical outcomes and a standardized mean difference of 0.13 (range −0.16 to 0.36) in three RCTs reporting continuous outcomes [9]. A recent review of trials of PEMs for improving physician behaviour specifically in primary care settings did not show significant improvement in physician behaviour across included trials [10].

The uncertainty in the evidence leaves policy makers uncertain about the role of PEMs, at a time when closing evidence-to-practice gaps has become a more cost-effective investment of health system resources than developing new interventions [11].

Reviews cannot overcome limitations of the primary evidence: the small number of trials (of varying size) and methodological weaknesses (insufficient power to detect modest effects, unit of analysis errors). Thus, there is a need for a pragmatic [12], randomized controlled trial on the effect of PEMs on guideline adherence, conducted in real-world settings, on typical practitioners, taking into account group practices. Given the simplicity and low cost of PEM-based practice change programs, and the large population impact that even modest improvements may achieve when applied to all potential beneficiaries, a large trial is needed.

We developed and used four criteria to identify important evidence/practice gaps in Ontario primary care on which to test the impact of PEMs in a pragmatic trial: the gap is large and important to patients; it involves a common disorder; evidence-based practice is not constrained by structural or financial barriers; and process indicators exist that are measurable using the administrative datasets available to us.

The present study addresses these criteria by targeting first-line treatment of uncomplicated hypertension using a thiazide diuretic. This clearly qualifies as an important primary care gap involving a common disorder not constrained by structural or financial barriers and for which routinely collected administrative data could be used to evaluate an intervention. If the above-mentioned modest impact on clinical practice of PEMs is applied, then at the trivial cost of a letter to each physician, Ontario’s health care system stands to save over $1 million, at current levels of treatment of hypertension [2, 5], with correspondingly greater savings if identification and treatment of hypertension is improved. The Ontario Printed Educational Materials (OPEM) trial aimed to evaluate different forms of PEMs: long inserts compared to shorter bullet-pointed outserts and different forms of outserts. We hypothesized that active arms would be superior to control, that individually, different forms of PEMs would be similarly effective on prescribing thiazides, and would be more effective when combined and when developed using a theory of behaviour.

## Methods

### The interventions

*informed* was a free, peer-reviewed, evidence-based practice synopsis, mailed to nearly 15,000 primary care providers in Ontario from 1994 to January 2007 (when publication ceased). Articles were developed by clinical and research staff from the Institute for Clinical Evaluative Sciences (ICES).

Two types of PEMs addressed the identified evidence-practice gap: a two-page article, indistinguishable from the rest of the newsletter in size and style (the “insert”) and two versions of a short, directive, evidence-based PEM on a postcard-sized card stapled to the front page of *informed* (the “outsert”). The insert and one of the versions of the outsert (the atheoretical outsert) were developed without any explicit theory of action using input from a diverse group of physicians who identified barriers to evidence-based practice and from a communications expert. The second outsert (the theory of planned behaviour-based outsert) was developed by a group consisting of three health psychologists and two implementation researchers with experience using theories of behaviour. Full details about the development process for this outsert are described in the study protocol [13]. The addition of the “theory of planned behaviour-based” outsert allowed us to test the hypothesis that a message inspired by a psychological theory, specifically the *theory of planned behaviour* [14] (TPB), would be more effective in changing clinical behaviour toward more evidence-based practice than a message designed without an explicit theoretical basis. The study design is shown in Table 1. Table 2 shows the two versions of the outserts, and the insert is included in Additional file 1. The interventions were included with the July 2005 edition of *informed*.

**Table 1:** Study design and number of practice groups/number of physicians

	Randomized		Included in the analysis	
			(started at least one patient with uncomplicated hypertension on medication during the follow-up year)	
Intervention	Number of practice groups	Number of physicians	Number of practice groups	Number of physicians
1. *informed* only (no PEM)	1057	1330	947	1166
2. *informed* plus insert	1058	1265	926	1093
3. *informed* plus outsert				
a. Atheoretical outsert	529	644	475	565
b. TPB-based outsert	529	652	476	585
4. *informed* plus insert and outsert				
a. Atheoretical outsert	529	648	461	550
b. TPB-based outsert	529	640	449	545
Total	4231	5179	3734	4504

Physician practices were randomly assigned to one of four intervention groups. The two intervention groups selected to receive an outsert were further randomly divided into two sub-groups, one of which received the outsert developed by the OPEM team (atheoretical outsert), the other receiving the TPB-based outsert. Interventions were included in the July 2005 edition of *informed*

*PEM* printed educational message, *TPB* theory of planned behaviour

**Table 2:** Theory of planned behaviour and atheoretical outserts

	Content of atheoretical message	Content of theory of planned behaviour-based message
Message wording	*Take a new look at THIAZIDES for first-line treatment for hypertension*	*Prescribe thiazide diuretics as the first drug to treat patients with hypertension*
	✓ BP control equal to all other antihypertensives	✓ You will be *more effective* in lowering your patients’ heart failure risk than if you prescribe calcium channel blockers
	✓ Better stroke prevention than ACE inhibitors	✓ You will be *more effective* in lowering patients’ stroke risk than if you prescribe ACE inhibitors
	✓ Better heart failure prevention than calcium channel blockers	✓ You can *feel good* about giving your patients the most effective treatment
		✓ You will be prescribing one of the most effective drugs as recommended by the *Canadian Hypertension Education Program*
	*Make THIAZIDES the first-line choice for YOUR patients*	Will YOU routinely prescribe thiazide diuretics? *YES* no
Attributes specified in study protocol		
Banner	Take a new look at THIAZIDES for first-line treatment for hypertension (11 words)	Prescribe thiazide diuretics as the first drug to treat patients with hypertension (12 words)
Up to four bullet points	✓ (3 bullet points)	✓ (4 bullet points)
Up to 85 words	✓ (40 words)	✓ (85 words)
Key clinical messages with footnotes on back of card	✓	✓
Cite the ALLHAT trial as evidence base for the recommended behaviour	✓	✓

### Data sources

Ontario has a single-payer public health insurance system, in which necessary medical and hospital care and prescription drugs are covered for all Ontario residents aged 65 years and older. The following administrative data sources from this insurance plan were used [15, 16]:

- The OHIP Claim History Database details payments to health care professionals, including an encoded provider number unique to each health care professional, an anonymous, encoded patient identifier unique to each patient, the service provided and the service date.
- The Canadian Institute for Health Information (CIHI) Discharge Abstract Database (DAD) contains the primary and up to 24 secondary diagnoses for all discharges from acute care hospitals.
- The OHIP Registered Persons Database (RPDB) contains basic demographic, place of residence and vital status information for each insured person.
- The OHIP Corporate Provider Database contains demographic and practice information for each physician.
- The Ontario Drug Benefit (ODB) Program database contains prescription drug claims data for eligible beneficiaries (age over 65, patients on social assistance and qualifying for Trillium [low income] drug program) of the program, including an encoded prescriber identifier.

Records from these datasets were linked, using unique, encoded patient and physician identifiers and analysed at the Institute for Clinical Evaluative Sciences (ICES; www.ices.on.ca), to determine which individuals were newly treated for hypertension during the study period, and which of the target physicians prescribed their antihypertensive medication.

### Study practices

All Ontario physicians with an active general/family practice in Ontario in 2003/2004 were eligible for inclusion. “Active” practice was defined as having a total billing volume for the year of at least $50,000 and writing prescriptions for at least 100 different patients (aged 65 years and older), with at least one prescription in at least 10 of the 12 months.

Physician identifiers were linked to the College of Physicians and Surgeons of Ontario (CPSO) number at ICES. CPSO numbers were then hand-linked to the publicly available CPSO database (www.cpso.on.ca) to obtain practice addresses.

Trials of interventions aimed at changing clinical practice must be randomized at the level at which they are directed—in this case, the family physician. In a group practice, doctors may share information. To prevent contamination, we randomized at the level of the practice. Physicians were placed into practices on the basis of a shared address.

### Study patients

The Ontario Drug Benefit database was used to identify Ontario residents who filled one or more prescriptions for an antihypertensive medication between July 11, 2005, and July 24, 2006. To ensure that these individuals were started on an antihypertensive agent for treatment of hypertension and not another indication, the cohort was linked with the CIHI DAD, the RPDB and the ODB Database. We excluded those patients with OHIP claims within 3 years or CIHI claims within 4 years (or any antihypertensive medications prescribed within 1 year) for any of the following conditions: myocardial infarction or angina, heart failure, arrhythmias, renal disease (including nephropathy), liver disease (including oesophageal varices), stroke or transient ischemic attack, hyperthyroidism or migraines [17]. Consistent with guideline recommendations for treatment of hypertension [18], we focused on thiazide prescribing as the first-line treatment in individuals with uncomplicated hypertension. In order to ensure that the cohort included only individuals newly treated for hypertension, individuals who had filled a prescription for any antihypertensive medication in the year prior to the intervention were also excluded [17]. Only the first prescription for an antihypertensive filled during the observation year was included; all prescriptions filled on the same day were taken into consideration when determining the outcome. While data were available for individuals 65 years and over, all individuals included in the study were at least 66 years old at the time they filled their first prescription for an antihypertensive medication in order to ensure that information covering a 1-year look back period for prior prescriptions was available.

Using the OHIP physician claims database, we identified the physician seen during the 14 days prior to and including the date the antihypertensive prescription was filled. In some cases, more than one study physician was selected. (Patients who had not visited any of the study physicians during the 14-day period were not included in the analysis.) We included patient visits to the physician’s office, physician visits to the patient’s home or to a patient in a long-term care facility and physician phone calls to the patient.

### Study design

The study was a pragmatic, factorial, cluster-randomized controlled trial with the physician practice as the unit of randomization. Practices were randomly assigned to one of six intervention groups by the study statistician (see Table 1), using computer-generated random numbers. Patient and physician participants were unaware of allocation and administrative data were collected without knowledge of the research under way. Full details of the study design can be seen in the published protocols [13, 19, 20].

### Outcomes

The objectives of the study were to determine whether the format of the PEM affected the likelihood that the only medication initially prescribed for hypertension was a thiazide and whether a theory of planned behaviour-based approach to developing the message was any more effective than a PEM developed without use of explicit theory. A “successful” outcome was a first-line prescription for a thiazide and no other antihypertensive medication. Combinations of a thiazide plus potassium or a potassium-sparing medication were counted as a “success”; combinations of a thiazide plus another diuretic were counted as a “failure”. A prescription for any non-thiazide antihypertensive medication was also counted as a “failure”.

### Power

Even a 5 % improvement in the prevalence of care for common conditions represents a meaningful health benefit. Based on pilot data, Monte Carlo simulations, assuming an intracluster correlation coefficient of 0.092, three patients per physician, a baseline success rate of 0.36 and absolute intervention effects of 0.05, 0.075 and 0.10, demonstrated that a trial with 1250 practices per arm would provide over 97 % power to detect a 5 % increase, and over 98 % power to distinguish between the effects of the combined intervention and either alone, assuming the combined effect to be additive. This original power calculation was based on testing the effectiveness of the insert and one version of the outsert. We conducted a follow-up simulation to assess whether we could test a second version of the outsert (theory of planned behaviour-based), which suggested that the power would be greater than 80 %. The research opportunity that this modification presented was judged to outweigh the loss of power. These decisions were made during the design phase and prior to randomization, as detailed in our published protocols [13, 19, 20].

### Statistical analysis

Logistic regression was used to test the hypothesis that the intervention affected the choice of first-line medication for hypertension. While the unit of randomization was the physician practice, outcomes were measured at the individual patient level. The logistic regression model was estimated using generalized estimating equations (GEE) to account for the clustering of patients within physician practices [21]. This method of analysis allows for the inclusion of patient-level and physician-level covariates (e.g. patient age, physician age), while at the same time accounting for possible correlations amongst patient outcomes within a practice.

Analysis was on an intention-to-treat basis. As shown in Table 3, models were fit to test: insert and both outserts combined, and insert and two outserts split (theory of planned behaviour-based vs. atheoretical). These were fit using unadjusted (regressions 1a and 2a) models and models adjusting for patient- and physician-level covariates (regressions 1b and 2b). The full model in 1b included interaction terms between the intervention and each covariate, to determine whether the impact of the intervention depended on patient and/or physician characteristics.

**Table 3:** Results of the logistic regression

Regression model 1a: unadjusted effect of interventions, insert and combined outserts			
Intervention	Odds ratio	95 % confidence interval	*p* value
*informed* only (reference group)	1.00		0.54
+ insert	0.97	0.86 to 1.09	
+ outsert	0.93	0.83 to 1.05	
+ insert and outsert	1.01	0.90 to 1.14	
Regression model 1b: effect of interventions, adjusted for patient and physician covariates, insert and combined outserts^a^			
Intervention	Odds ratio	95 % confidence interval	*p* value
*informed* only (reference group)	1.00		0.60
+ insert	0.98	0.87 to 1.11	
+ outsert (combined)	0.93	0.83 to 1.05	
+ insert and outsert	1.00	0.89 to 1.12	
Effect of patient and physician characteristics^b^			
*Physician characteristics*			
Female (reference is male)	1.27	1.14 to 1.40	<0.0001
*Place of training*			<0.0001
Canada (reference)	1.00		
USA	0.90	0.77 to 1.06	0.21
UK, Ireland, Australia, New Zealand	0.75	0.43 to 1.30	0.30
Other	0.53	0.46 to 0.61	<0.0001
Group practice (reference is solo practice)	1.21	1.10 to 1.32	<0.0001
Rural (reference is non-rural)	1.39	1.23 to 1.57	<0.0001
Years since graduation (odds ratio per additional 10 years)	1.06	1.01 to 1.11	0.014
Elapsed time between mail-out and patient visit (odds ratio per additional 30 days)	0.98	0.97 to 0.99	<0.0001
*Patient characteristics*			
Female sex (reference is male)	1.37	1.29 to 1.45	<0.0001
Age (odds ratio per additional 10 years of age)	1.09	1.05 to 1.14	<0.0001
*Location of the visit*			<0.0001
Physician office (reference)	1.00		
Long-term care	0.68	0.56 to 0.84	0.0002
Patient’s home	0.96	0.68 to 1.35	0.81
Phone call	0.30	0.13 to 0.70	0.0050
Regression model 2a: unadjusted effect of interventions, with outserts split by type			
Intervention	Odds ratio	95 % confidence interval	*p* value
*informed* only (reference group)	1.00		0.69
+ insert	0.97	0.86 to 1.09	
+ atheoretical outsert	0.95	0.82 to 1.09	
+ TPB-based outsert	0.92	0.80 to 1.06	
+ insert and atheoretical outsert	0.97	0.84 to 1.12	
+ insert and TPB-based outsert	1.05	0.91 to 1.21	
Regression model 2b: effect of interventions, adjusted for patient and physician covariates, insert and outserts split by type			
Intervention	Odds ratio	95 % confidence interval	*p* value
*informed* only (reference group)	1.00		0.71
+ insert	0.98	0.87 to 1.11	
+ atheoretical outsert	0.94	0.81 to 1.08	
+ TPB-based outsert	0.93	0.81 to 1.07	
+ insert and atheoretical outsert	0.96	0.83 to 1.10	
+ insert and TPB-based outsert	1.05	0.91 to 1.21	

^a^The model was adjusted for these patient variables: age, sex and location of the visit with the physician. The model was adjusted for these physician variables: year of graduation, sex, place of training, type of practice (solo/group), place of practice (rural/urban) and elapsed time between the mail-out and the office visit

^b^Odds ratios are adjusted for all of the other variables in the model

*p* values for interactions with the intervention

Informed subscriber *p* = 0.73

Rural location *p* = 0.97

Practice type, *p* = 0.88

Patient sex, *p* = 0.81

Location of visit, *p* = 0.76

Patient age, *p* = 0.58

GP sex, *p* = 0.36

Number of years since GP graduation, *p* = 0.29

Graduation place, *p* = 0.18

Time from mail-out to patient visit, *p* = 0.15

*p* value for four-level intervention = 0.60

*p* value for six-level intervention = 0.71

All of the main effects are significant

Patient and physician characteristics were compared between randomization arms using chi-square tests for categorical variables and Kruskal-Wallis tests for continuous variables.

All analyses were performed at ICES using SAS version 9 (SAS Institute, Cary, North Carolina). Two-tailed *p* values less than or equal to 0.05 were considered to be significant.

## Results

### Physician and patient selection

**Fig. 1:**
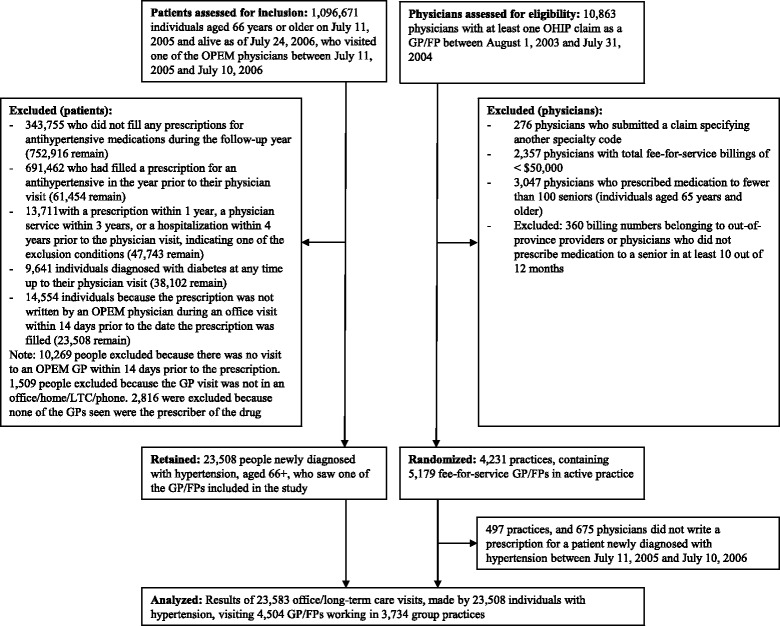
Patients and physicians included

Figure 1 shows the number of physicians and patients included in the study. Three quarters of Ontarians aged 66 years and older filled a prescription for an antihypertensive medication in the year following the mail-out, but almost all (92 %) had already filled a prescription for an antihypertensive medication during the preceding year. A further 38 % of those who filled a prescription for the first time were excluded because there was evidence that the antihypertensive might have been prescribed for a reason other than hypertension. We identified 38,102 individuals who were taking medication for hypertension for the first time and were able to link the prescriptions of 23,508 of these individuals to one of the physicians targeted by the intervention (Fig. 1). Thirteen percent of family physicians randomized to receive one of the interventions were excluded from the analysis because they were not linked to at least one patient newly treated for hypertension.

### Physician and patient characteristics

There were small, statistically significant but clinically unimportant, differences between the characteristics of the physicians in the six intervention groups (Table 4).

**Table 4:** Physician and patient characteristics, by intervention group

	*informed* only	Insert	Atheoretical outsert	TPB-based outsert	Insert + atheoretical outsert developed	Insert + TPB-based outsert	All	*p* value*
	*N* = 1166	*N* = 1093	*N* = 565	*N* = 585	*N* = 550	*N* = 545	*N* = 4504	
Physician characteristics								
Sex (% male)	76.3	77.5	75.4	80.5	78.9	76.0	77.3	0.25
Place of training (%)								0.24
• Canada or USA	78.2	75.6	73.8	78.6	77.8	80.8	76.8	
• UK Ireland, Australia, New Zealand	7.8	8.8	11.2	9.4	11.6	8.1	9.2	
• Other	14.0	15.6	15.0	12.0	10.6	11.1	14.0	
Solo practice (%)	66.1	72.7	70.3	68.0	69.5	68.6	69.2	0.030
Rural^a^ (%)	11.0	12.1	14.9	12.5	12.7	10.3	11.9	0.18
Years since graduation: mean (std)	26.3 (10.2)	27.0 (10.1)	26.7 (10.3)	26.9 (10.0)	26.4 (10.0)	26.5 (9.2)	26.6 (10.0)	0.51
Elapsed time between mail-out and patient visit (days): median (25th, 75th percentiles)^b^	185 (99, 276)	191 (105, 275)	196 (103, 275)	190 (102, 274)	184 (99, 270)	186 (101, 274)	189 (101, 274)	0.26
Patients newly treated for hypertension started on only a thiazide (%)	27.6	27.4	27.3	26.3	27.8	28.9	27.5	0.69
Patient characteristics								
Number of patient visits	6151	5507	3028	3188	2907	2802	23,583	
Sex (% female)	53.0	52.6	54.5	53.4	51.8	52.1	52.9	0.32
Age at time of visit to physician: median (25th, 75th percentiles)	74 (70, 79)	74 (70, 79)	74 (70, 79)	74 (70, 80)	74 (70, 80)	74 (70, 79)	70 (70, 79)	0.25
Location of the visit (%)								0.051
• Physician office	95.4	95.7	95.5	95.9	94.8	95.9	95.5	
• Long-term care	3.5	3.2	3.5	3.0	4.5	3.0	3.4	
• Patient’s home or consultation by phone	1.2	1.0	1.0	1.1	0.7	1.2	1.0	

*p value testing the null hypothesis that there was no difference amongst the intervention groups. The proportion of patients receiving an eye exam was compared using GEE

^a^A practice area was designated as rural if it was located in a geographic region with a population smaller than 10,000

^b^Elapsed time from the date of the mail-out to the date of the patient’s visit was measured for each patient rather than for each physician

### Analysis of intervention effects

The intracluster correlation coefficient was 0.18 (95 % confidence interval 0.16 to 0.19). Intervention effects are shown in Table 3. Neither the unadjusted nor the adjusted results show evidence that any of the interventions, alone or in combination, were effective. The widest confidence interval reported in the table, an odds ratio between 0.91 and 1.21 for the insert + TPB-based outsert corresponds to a true absolute effect of the intervention lying between a decrease of 1.5 % and an increase of 3.5 %. Thus, not only did the intervention fail to achieve statistical significance but as well the confidence interval does not contain values of much practical importance.

While the probability of being prescribed a thiazide as the first-line drug depended on both physician and patient characteristics, there was no indication that the interventions themselves were any more or less effective in any physician or patient sub-group. The *p* values for the interactions between the intervention and the physician/patient variables were all non-significant, ranging from 0.15 to 0.97.

## Discussion

This printed information intervention was designed to increase physician prescribing of thiazides as the first-line pharmaceutical treatment for hypertension. The interventions, evaluated in a very large trial, with sufficient power to detect a small change in physician behaviour, failed to change prescribing practice. This confirms the results of studies [22] that found no impact of mailing the Ontario hypertension guidelines to all physicians in Ontario.

Given the pragmatic and representative nature of our study, we propose that these results may apply also to primary care practitioners in other health care settings with universal health insurance and no cost to patient for drugs.

This is the first published trial reporting a head-to-head comparison of TPB-based vs. atheoretical implementation interventions. Although this trial did not detect any difference in effectiveness between these two approaches to intervention, this question needs further research.

We found that only 27.5 % of the individuals newly started on antihypertension medication were started on only a thiazide. This is similar to the rate of 29 % reported by Morgan et al. [3] for another Canadian jurisdiction (although Morgan et al. included patients whose first hypertension treatment was a thiazide diuretic along with another antihypertensive drug, in addition to those who received only a thiazide diuretic) but lower than the 35 % rate reported for Ontario between 1994 and 2002 [17].

Female patients and older patients were more likely to be prescribed thiazides as first-line treatment, corresponding to patterns observed elsewhere [3, 17, 23]. Female physicians, physicians who had been in practice longer, physicians in group practices and physicians practicing in rural locations were more likely to prescribe thiazides as first-line treatment (Table 3).

Depending on the condition being treated, improvements in prescribing patterns have the potential to save patients/insurers money as well as improving patient outcomes. It is therefore important to pursue other ways of changing prescribing practices. Several studies report that a combination of prescribing feedback plus educational intervention is effective in increasing the rate of thiazide prescribing [24–26]. The Canadian Hypertension Education Program (CHEP) has been able to produce sustained improvements in the clinical management of hypertension by combining annual updates of its recommendations with an extensive implementation program that includes both passive and active dissemination, including workshops and academic detailing [27].

An economic analysis found that if the initial improvements in practice can be sustained, they may be cost-effective [2, 28]. Furthermore, cost-effectiveness of these interventions should improve as changes in patient demographics mean that physicians are likely to see increasing numbers of patients with hypertension in their practices.

A strength of our study was the use of *informed* as the vehicle carrying the PEMs into physician offices. The effectiveness of printed educational materials depends, firstly, on whether they are read. In 1997, The Strategic Council Inc. contacted 500 Ontario physicians by phone to determine readership and recall of *informed*. They found that 71 % of the respondents recalled receiving *informed* and that of these, 89 % found it useful or very useful and 53 % read most or every issue [29]. Two surveys of *informed* subscribers, conducted in 1995 and 1999, found that the newsletter was a respected and valued source of information [29]. It is unlikely, then, that the failure of this study to change outcomes was related to the perceived trustworthiness of the source or the failure of the physicians to notice the messages.

Another strength of the study was the use of administrative data, which allowed us to examine the impact of our interventions across the full spectrum of physicians and patients in Ontario. This strength also imposes some limitations, one of which is the inability to study non-fee-for-service physicians. However, it is estimated that only 2 % of Ontario primary care physicians were on alternative payment plans whose billings did not appear in OHIP claims at the time of the study [30].

A second limitation imposed by reliance on administrative data is that we cannot differentiate between failure of the PEM to be delivered, read or remembered, failure of the physician to advise the patient and failure of the patient to act on that advice. While the Canadian postal service is highly reliable, and the addresses used are equally so, it is possible that, despite the widely recognized brand of *informed* as an evidence-based newsletter from a respected research institute (rather than a product marketing leaflet), it may not have been received by study physicians. This is a possible fate for all PEMs and so does not detract from our main conclusion that PEMs do not change practice.

From the perspective of patient health, interventions are useful only if they affect the treatment the patient receives. This was a pragmatic trial, designed to give a definitive answer about the value of a particular mode of information transmission for the purpose of improving health care. The trial was not designed to explain the barriers that remained. We did however investigate the reasons that may explain the lack of observed effect in a theory-based process evaluation conducted alongside the trial [31].

## Conclusions

Consistent with systematic review findings [9], this study supports the conclusion that PEMs, whether long and discursive, or short and directive, and whether based on a theory of behaviour or no theory at all, did not, on their own, bring about an effective change in physician prescribing, even in the absence of financial and structural barriers to change.

## Additional file

Additional file 1: Under pressure: the latest on managing hypertension. (PDF 291 kb)
