# Supplementary material for: Printed educational messages fail to increase use of thiazides as first-line medication for hypertension in primary care: a cluster randomized controlled trial [ISRCTN72772651]
Source: Implement Sci. 2016 Sep 17;11:124. doi: 10.1186/s13012-016-0486-3 (PMC5027087; doi:10.1186/s13012-016-0486-3)
Supplement: Additional file 1: — Under pressure: the latest on managing hypertension. (PDF 291 kb) [file 13012_2016_486_MOESM1_ESM.pdf]

informed Xtra

JULY 2005 VOL 11 NO 3

Under Pressure

The latest on managing hypertension

The Canadian Hypertension Education Program (CHEP) recently released their 2005 Canadian Recommendations for the Management of Hypertension (http://www.hypertension.ca). The CHEP recommendations are updated annually according to an evidenced-based review of the latest international research. Although there have not been any recent studies on how well we are doing in detecting and managing hypertension, previous research suggests that there is a lot of room for improvement. Around 43% of people who have hypertension do not know they have it. Only 13% of those with hypertension are both treated *and* controlled.

STEP 1 Diagnosis

Previous guidelines recommended up to six office visits over a six-month period before making a diagnosis of hypertension. Recognizing the impracticability of this process, the CHEP task force has outlined new criteria for diagnosing hypertension with the hope of reducing unnecessary delays in diagnosis and treatment. Office, ambulatory or home measurements can be used to diagnose hypertension.

The new criteria are as follows:

- **1 Visit:** Hypertension is diagnosed immediately in hypertension emergencies or urgencies.
- **2 Visits:** Sustained blood pressures ≥180/110 mm Hg *or* sustained blood pressures ≥ 140/90 mmHg in the presence of diabetes, chronic kidney disease, or target organ damage (affecting brain, heart, eyes, kidneys and peripheral arteries).
- **3 Visits:** Sustained blood pressure ≥160/100 mm Hg.
- **5 Visits:** Sustained blood pressure ≥ 140/90 mm Hg.
- **Home/self measurement:** Duplicate home readings in the morning and evening for one week (excluding day 1) ≥135/85 mm Hg.
- **Ambulatory blood pressure monitoring:** Average daytime pressure ≥135/85 mm Hg or 24 hour average ≥130/80 mm Hg.

A diagnosis of hypertension can be made if **either** the systolic or diastolic blood pressure is elevated. Although CHEP has added criteria for home and ambulatory blood pressure measurement, the authors caution that these readings must be done on internationally validated equipment. Assessment of self-measurement blood pressure devices can be found at http://www.dableducational.com/sphygmomanometers/devices_2_sbpm.html.

STEP 2 Initial investigations

Patients diagnosed with hypertension should have the following laboratory tests:

- Urinalysis.
- Complete blood count.
- Blood chemistry (sodium, potassium, creatinine).
- Fasting blood glucose.
- Fasting total cholesterol, high density lipoprotein (HDL), low density lipoprotein (LDL), triglycerides.
- Standard 12-lead electrocardiogram.

Further investigations should be guided by the laboratory results, physical examination and history.

STEP 3 Risk assessment

A thorough cardiovascular (CV) risk assessment is important in managing patients with hypertension, as over 90% of patients with hypertension have other CV risks. It is useful in managing the identified risk factors, as well as in choosing specific drug therapies and target blood pressures. Simply adding together risk factors underestimates a person's risk. Although there are several algorithms available, CHEP endorses the WHO/ISH evaluation of risk based on hypertension readings and CV risk factors (see table below).

Cardiovascular risk factors include:

- ✔ Male gender.
- ✔ Increasing age.
- ✔ Previous stroke or transient ischemic attack (TIA).
- ✔ Microalbuminuria or proteinuria.
- ✔ Diabetes mellitus.
- ✔ Smoking.
- ✔ Family history of premature CV disease (age ≥45 years for men; age ≥55 years for women).
- ✔ Left ventricular hypertrophy.
- ✔ Total cholesterol to HDL (high density lipoprotein) ratio ≥6 in men, ≥5 in women.
- ✔ Chronic kidney disease (glomerulofiltration rate <60 mL/min/1.73m^2).
- ✔ Sedentary lifestyle.

STEP 4 Target blood pressure

It is important to treat to target blood pressures. These targets vary depending on associated conditions (see table below).

Target values for blood pressure

Condition	Target blood pressure
Diastolic +/- systolic hypertension	<140/90
Isolated systolic hypertension	<140
Diabetes	<130/80
Renal disease	<130/80
Proteinuria >1 g/day	<125/75

Adapted from: CHEP. Canadian Recommendations for the Management of Hypertension. 2005.

Association of blood pressure, risk factors and target organ damage

	Blood Pressure (MM HG)				
Other risk factors and disease history	Normal (SBP 120–129 or DBP 80–84)	High normal (SBP 130–139 or DBP 85–89)	Grade 1 (SBP 140–159 or DBP 90–99)	Grade 2 (SBP 160–179 or DBP 100–109)	Grade 3 (SPB > 180 or DBP >110)
I. No other risk factors	Average risk	Average risk	Low added risk	Moderate added risk	High added risk
II. 1–2 risk factors	Low added risk	Low added risk	Moderate added risk	Moderate added risk	**Very high** added risk
III. ≥ 3 risk factors *or* target organ damage *or* diabetes	Moderate added risk	High added risk	High added risk	High added risk	**Very high** added risk
IV. Associated clinical conditions	High added risk	**Very high** added risk	**Very high** added risk	**Very high** added risk	**Very high** added risk

Adapted from: CHEP. Canadian Recommendations for the Management of Hypertension. 2005. SBP=systolic blood pressure. DBP=diastolic blood pressure.

Continued ▸ 2

Impact of lifestyle therapies on the treatment of hypertension

Therapy	CHEP recommendations	Change in systolic BP/diastolic BP
**Healthy diet.**	DASH diet.*	-11.4/-5.5
**Regular physical activity.**	Moderate exercise (30–60 min/day) ≥4 times/week.	-7.4/-5.8 *(based on exercise 3 times/week)*
**Reduction in alcohol intake in those who drink excessively.**	≤2 drinks/day (maximum 14/week for men; 9/week for women).	-4.6/-2.3 *(based on reduction of 2.7 drinks/day)*
**Weight loss in those who are overweight.**	BMI <25 kg/m^2.	-7.2/-5.9 *(based on weight loss of 4.5 kg)*
**Waist circumference.**	<102 cm for men. <88 cm for women.	
**Salt restriction in individuals considered salt sensitive** (e.g. African descent, age over 45, individuals with impaired renal function or diabetes).	65–100 mmol/day.	-5.8/-2.5 *(based on reduction of 100 mmol/day)*
**Smoking cessation.**	Smoke free environment.	

Adapted from: CHEP. Canadian Recommendations for the Management of Hypertension. 2005. *For more information on DASH diet, see http://www.nhlbi.nih.gov/health/public/heart/hbp/dash/new_dash.pdf.

STEP 7 Combination therapy

Over 60% of patients with hypertension will require at least two or more drugs to achieve target blood pressures. The CHEP recommendations suggest some useful combinations (see table below).

Useful antihypertensive drug combinations

Column 1	Column 2
• Thiazide diuretics<br>• Long-acting calcium channel blocker*	• Beta-blocker<br>• ACE inhibitor<br>• Angiotensin II receptor blocker
*For dual therapy, combine any agent from **column 1** with any in **column 2**.*	*For triple therapy, combine two agents from one column with any agent from the other column.*

Adapted from: CHEP. Canadian Recommendations for the Management of Hypertension. 2005. *Caution should be exercised when combining a non-dihydropyridine calcium channel blocker with a beta blocker.

STEP 5 Lifestyle modifications

All patients with hypertension should be counseled on lifestyle modification. Changes in lifestyle have been shown to reduce blood pressure (see table above).

STEP 6 First-line drug therapy

CHEP recommends that pharmacotherapy be *strongly* considered if the average diastolic blood pressure is:

- ≥80 in a person with diabetes.
- ≥90 in a person with hypertensive end organ damage/CV disease *or* independent CV risk factors.

The 2005 CHEP recommendations emphasize that reducing hypertension-associated complications in the "general" population of those with hypertension is more dependent on the extent of blood pressure lowering than on the choice of drug. Based on efficacy data alone, they endorse five categories of antihypertensive agents as suitable first-line therapy. See table right with drug categories and costs.

ALLHAT was an international landmark randomized controlled trial comparing amlodipine, chlorthalidone, doxazosin and linsinopril in preventing fatal coronary artery disease or nonfatal myocardial infarction in high-risk patients with hypertension. During the trial, the doxazosin arm was dropped due to a 25% higher rate of combined CV outcomes. In the end, they found no difference in chlorthalidone compared to amlodipine or lisinopril in preventing major coronary events or increasing overall survival. The authors concluded that thiazide-type diuretics should be the drugs of choice for first-line therapy in uncomplicated hypertension because of their superior prevention of CV disease and lower cost (see box left for comparative costs).

Comparative costs of recommended first-line therapies for hypertension

Drug category	Average monthly cost in CDN $ (excluding dispensing fee)
**Thiazide diuretics**	**$4**
**Beta-adrenergic blockers**	**$13–$38**
**Angiotensin-converting enzyme inhibitors**	**$26–$41**
**Angiotensin II receptor blockers**	**$45–48**
**Long-acting calcium channel blockers**	**$31–$75**

Adapted from: http://www.rxfiles.ca/acrobat/cht-HTN-1page%20summary.pdf (accessed May 10, 2005.)

When choosing a first-line medication for an individual patient, associated risk factors, target end-organ damage/complications and concomitant diseases/conditions should be considered. For more information on individualization of therapy, see http://www.hypertension.ca/recommendations_2005/execsummary2005.pdf.

STEP 8 Additional considerations

A few other key points in the management of hypertension:

- Consider low-dose acetylsalicylic acid in your patients with controlled hypertension.
- Consider a statin in patients with hypertension who have established CV disease or the presence of three or more CV risk factors including age ≥55 years and male gender.
- Adherence to therapy should be assessed at each visit. Medication regimes should be simplified and tailored to fit patients' daily habits.

The Bottom Line

- **The diagnosis of hypertension can be expedited and include office, home or ambulatory blood pressure measurements.**
- **Thiazide diuretics (e.g. hydrochlorothiazide) should be the drugs of choice for first-line therapy, as they are both effective and inexpensive.**
- **Many patients will require at least two drugs to achieve their target blood pressures.**

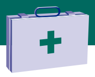

Article Peer-reviewed

Institute for Clinical Evaluative Sciences (ICES), G1 06, 2075 Bayview Avenue, Toronto, ON M4N 3M5
Phone: (416) 480-4055, Extension 5168 Internet: http://www.ices.on.ca E-mail: informed@ices.on.ca
